# The Microbiome in Periodontitis and Diabetes

**DOI:** 10.3389/froh.2022.859209

**Published:** 2022-04-08

**Authors:** Davi Neto de Araújo Silva, Maísa Casarin, Sepehr Monajemzadeh, Beatriz de Brito Bezerra, Renate Lux, Flavia Q. Pirih

**Affiliations:** ^1^Section of Periodontics, School of Dentistry, University of California, Los Angeles, Los Angeles, CA, United States; ^2^School of Dentistry, Federal University of Pelotas, Pelotas, Brazil

**Keywords:** periodontitis, diabetes mellitus, oral microbiome, genetics, integrative review

## Abstract

**Objectives:**

To perform a comprehensive and integrative review of the available literature on the potential changes in the microbiome of healthy and individuals with diabetes under periodontal health and disease.

**Materials and Methods:**

The review was conducted by two independent reviewers. Indexed electronic databases (PubMed/Medline, Cochrane Library, Web of Science and Scopus) were searched, including articles published in English and dated from 5 years ago until December 2021. A manual search also was performed to identify co-related articles. Following the removal of duplicates and eligibility criteria, the articles were included in tables for analysis and described in the manuscript.

**Results:**

According to this review, diabetes mellitus was associated with significant changes in the subgingival and salivary microbiome, either in its association with periodontitis or in cases of periodontal health. In addition to affecting microbial diversity in terms of taxonomy, metagenomic studies have shown that this endocrine disorder may also be directly related to increased pathogenicity in the oral microbiome.

**Conclusion:**

Although the reviewed studies demonstrate important differences in the subgingival and salivary microbiome composition because of diabetes mellitus, further studies are needed to clarify the real effects of hyperglycemia on oral microbial profiles and support new diagnostic approaches and innovative treatments.

## Introduction

Periodontitis is a chronic multifactorial inflammatory disease associated with dysbiotic plaque biofilms and characterized by the progressive destruction of the tooth-supporting apparatus [1]. Diabetes mellitus is a group of metabolic diseases characterized by abnormal insulin secretion, insulin action, or both situations leading to hyperglycemia [2]. Inflammation plays a central role in periodontitis and diabetes mellitus [3]. Both diseases have a high epidemiological impact worldwide with periodontitis affecting nearly 750 million people [4–6], and an estimated 415 million adults aged 20–79 with diabetes mellitus, including 193 million who are undiagnosed [2, 7–9].

Although there are no phenotypic features unique to periodontitis in patients with diabetes mellitus, diabetes mellitus increases periodontitis risk. In addition, periodontitis affects glycemic control and its complications [10–13]. This “two-way” relationship between periodontitis and diabetes mellitus has stimulated the development of several clinical and experimental studies focusing on identifying the molecular pathways that link these two conditions and understanding how they may potentially affect each other [8, 10, 13–15]. In general, periodontitis effects on diabetes mellitus may be related to bacteremia and/or the presence of inflammatory mediators in the systemic circulation. Consequently, an exaggerated systemic inflammatory response to subgingival bacteria leads to an acute-phase protein burst and, systemically, high levels of pro-inflammatory mediators that facilitate insulin resistance [16–18].

Based on this, it is assumed that the subgingival microbiome plays a fundamental role not only in the periodontitis pathogenesis, as has been reported in several taxonomic identification studies [19–25], but also in the bidirectionality with diabetes mellitus [26, 27]. Herein, we aim to better understand the diabetes mellitus impact on the oral microbiome by reviewing recent studies that analyzed the oral microbiome in individuals with periodontal health, periodontal disease, and diabetes.

## Methodological Aspects

This work is a literature review on the oral microbiome and its variations in individuals with diabetes mellitus in periodontal health and disease. The searches were performed by two independent reviewers in the main international databases (PubMed, Medline, Cochrane Library, Web of Science, and Scopus), in addition to a manual search. The search strategy used in all databases included the descriptors and MeSH (medical subject headings) terms “(periodontitis) AND (diabetes) AND (oral microbiome) AND (genetics)” without study design distinction. The analyzed articles were published in English and spanned the past 5 years. A total of 37 studies published between 2016 and 2021 were evaluated after the initial electronic search. After reading all the publications, 26 publications were excluded as they failed to mention the search terms, while 10 were fully evaluated and included in the presented discussion. A second search strategy was carried out, similar to the previous one, but excluding the last descriptor and adding the term “(salivary).” This generated a total of 16 articles, but only 8 matched the search criteria and were included.

## Key Principles Of The Oral Microbiome In Healthy Conditions And Periodontitis

The oral microbiome represents an important part of the human microbiota and includes more than a thousand species [28, 29]. As a human being develops from birth to adulthood, the oral microbiome changes, and succession mechanisms are observed [30, 31]. For example, there is a change in the predominance of *Escherichia coli, Staphylococcus*, and *Pseudomonas* present before tooth eruption to the predominance of *Fusobacterium, Prevotella*, and *Streptococcus mutans* linked to mature oral microbiomes [32–34].

Changes in the oral microflora have also been associated with systemic diseases such as diabetes mellitus as well as oral diseases such as periodontitis [35–37]. In this sense, as an important resource for phylogenetic, taxonomic, genomic, and phenotypic identification of the human oral microbiome [29], the expanded human oral microbiome database (eHOMD) [28] provides access to data on hundreds of cultivable and non-cultivable prokaryotic species [38, 39].

On that note, the microbial biofilm, the primary etiological factor in periodontitis, has been extensively studied and can include 500 species or more among cultivable and non-cultivable strains in a single person, with more than 800 species having been identified in different dental biofilms [40–42]. It is estimated that the total number of species may well-exceed 1,000, although most are non-cultivable [43–45].

Total bacterial levels in the subgingival environment vary according to periodontal conditions, with ~10^3^ in healthy shallow sites and 10^8^ in deep periodontal pockets, including putative pathogens, such as anaerobic gram-negative bacteria, spirochetes, fungi, and even viruses [46]. Gram-negative bacterial species such as *Porphyromonas gingivalis, Treponema denticola*, and *Tanerellaforsythia* have been systematically associated with the onset and progression of periodontitis in deeper anaerobic areas, while others dominated the gingival plaque composition in health [46].

Given this diversity, the microbial “complexes” concept emerged, showing that there is a shift in biofilm colonization from health to disease, as well as in the development and progression of periodontitis [43]. Over the last few decades, hypotheses such as the “non-specific plaque hypothesis,” [47] the “specific plaque hypothesis,” [48] the “ecological plaque hypothesis,” [49] and the “fundamental pathogen hypothesis” [50] helped to inform knowledge for future microbiological research on understanding the complex nature of the onset and progression of microbial diseases in the oral environment. Currently, OMICs approaches (e.g., genomics, transcriptomics, proteomics, and metabolomics) have increased the understanding of the oral microbial interactions, and it is now possible to identify all microbial species that colonize the mouth [43, 44].

## Subgingival Microbiome Affected By Diabetes Mellitus

Despite several studies focusing on the bidirectional relationship between diabetes mellitus and oral conditions such as periodontitis, the available literature still has limitations regarding the number and quality of studies addressing the impact of diabetes mellitus on the oral microbiome [51, 52].

Since glucose levels in the gingival crevicular fluid are similar to those in serum, the high availability of glucose may favor increased levels of saccharolytic commensals [53, 54], boosting the growth of fermenting species, including *Streptococcus anginosus, Filifactor alocis*, and generating a selective environmental pressure of glucose availability [55, 56].

In recent years, the development of next-generation sequencing technologies, as well as the Human Microbiome Project based on informative marker genes, community gene inventories (metagenomics), and functional analyses (metatranscriptomics), has contributed to the study of the human microbiome, including the oral microbiome and related systemic diseases [19, 22, 53, 54, 57].

Although some studies derived from high-throughput metagenomic sequencing of the oral microbiome have not been conclusive about the differences caused by diabetes mellitus [58–60], other studies with 16S rRNA gene deep sequencing have suggested that periodontally healthy individuals but, with diabetes mellitus, are at risk for periodontitis due to a decrease in the relative abundance and prevalence of species compatible with health (such as *Atopobium* and *Corynebacterium*) and an increase in the pathogenic content of the hyperglycemic microbiota (including *Porphyromonas, Prevotella, Campylobacter*, and *Fusobacterium*) [61, 62].

Despite its importance, 16S rRNA-based research is largely limited to taxonomic composition and only allows computational prediction analysis [63] of the microbial genomic potential of the studied communities [54, 64]. To date, metagenomic studies in the diabetes mellitus field have mainly focused on the gut metagenome [65–67]. However, using metagenomic shotgun sequencing to understand the susceptibility to periodontitis in the oral microbiome of individuals with diabetes, a recent study has identified distinct differences in the subgingival microbiome of patients with type 2 diabetes mellitus compared to non-diabetics. While the red complex species genes were less prevalent in the periodontitis state in diabetes mellitus compared to non-diabetics, in the healthy periodontal state, the subgingival microbiome in patients with diabetes mellitus contained more genes from orange complex species [27].

In another study, complete metagenomic shotgun sequencing of the subgingival microbiome showed that periodontitis was associated with a significantly higher relative abundance of oral taxon 439 of the bacterium *Anaerolineaceae* in patients with type 2 diabetes mellitus [68].

Applying 16s rRNA sequencing, Matsha et al. examined the bacterial composition in subgingival plaque samples from 128 patients with periodontitis and showed that *Fusobacteria* and *Actinobacteria* were significantly more abundant in subjects with type 2 diabetes mellitus, where the former increased the odds of diabetes mellitus by 14% and the latter increased the odds by 10%, both in subjects with gingival bleeding. However, according to the authors, it is not clear whether these differences were the consequence of hyperglycemia or the presence of periodontitis [52].

Corroborating with this, a consensus report from the American Academy of Periodontology and the European Federation of Periodontology [69] stated that there was no compelling evidence that diabetes mellitus significantly impacted the oral microbiota. This conclusion was based on several human studies that reported inconsistent and contradictory findings on whether diabetes mellitus altered the bacterial composition in the oral cavity [51]. However, some authors report evidence that type 2 diabetes mellitus reduces the diversity and richness of the subgingival microbiome, and this decrease is even associated with inadequate glycemic controls [56, 59, 70]. A summary of studies examined for this review is shown in Table 1.

**Table 1:** Subgingival microbiome affected by diabetes mellitus.

**References**	**Study design**	**Population**	**Test for microor** **ganisms' detection**	**Key microbiome**	**Glycemic parameters**	**Periodontal parameters**	**Diabetes type**	**Outcomes in diabetic groups**	**Conclusions**
Matsha et al. [52]	Case-control study	128	16S rDNA sequencing	*Firmicutes, Proteobacteria, Bacteroidetes, Fusobacteria, Actinobacteria, Spirochaetes, Synergistetes, Chloroflexi, and Tenericutes*	Plasma glucose and HbA1c	BOP, PD	2	↑*Firmicutes, ↑Proteobacteria, ↑Bacteroidetes, ↑Fusobacteria, ↑Actinobacteria*.(DM with gingival bleeding)↑*Bacteroidetes ↓Actinobacteria*	Oral microbiota changed in different glycemic statuses and stages of periodontal disease
Joaquim et al. [71]	Case-control study	100	Quantitative real-time PCR	*Porphyromonas gingivalis,Tannerella forsythia, Treponema denticola, Eubacteriumnodat, Parvimonas Micra, Fusobacterium nucleatum ssp., and Prevotella intermedia*	HbA1c, fasting plasma glucose	Visible plaque accumulation, BOP, marginal bleeding, PD and CAL	2	There were no differences between groups	Subgingival levels and bacterial prevalence are not significantly different in chronic periodontitis presenting DM, smokers, or smokers with DM.
Demmer et al. [70]	Cohort study	152	Human Oral Microbe Identification Microarray	*Actinobacteria and Proteobacteria*	HbA1c, fasting plasma glucose	BOP, PD, and CAL	2	18 taxa associated with inflammation. 22 taxa associated with insulin resistance	Inflammation: ↓*Actinobacteria ↓Proteobacteria ↑Firmicutes* and TM7
Saeb et al. [59]	Cross-sectional case-control	44	16S rRNA profiling	*-*	Fasting plasma glucose	PPDand CAL	2	↓Phylogenetic diversity	Pre-diabetic subgingival microbiota associated withreduced phylogenetic diversity
Long et al. [62]	Case-control study	294	16S rRNA gene sequencing	*Actinomyces* and *Atopobium*	History of diabetes	-	2	*↓Actinobacteria phylum*	The oral microbiome may play an important role in the diabetes etiology
Babaev et al. [72]	Cross-sectional	74	16s rRNA sequencing	*P. gingivalis, T. forsythia, A. actinomycetemcomitans, T. denticola, P. intermedia, F. nucleatum/periodontium, and P. endodontalis*	-	-	2	*↑Porphiromonadaceae, Fusobacteriaceae (Combined pathology) ↓Sphingobacteriaceae bacteria (Periodontitis)*	The metagenomic analysis confirmed the microbiota pathogenic role in combined pathology
Ganesan et al. [61]	Cross-sectional	100	16S rRNA gene sequencing	*Fusobacterium, Parvimonas, Peptostreptococcus, Gemella, Streptococcus, Leptotrichia, Filifactor, Veillonella, TM7 and Terrahemophilus*	HbA1c	CAL, PD, Mean gingival index	2	Diabetic microbiomes exhibited significant clustering based on HbA1c levels	Diabetics and diabetic smokerswere microbially heterogeneous and enriched for facultative species
Bachtiar et al. [73]	Cross-sectional	12	16S rRNA amplicon sequencing	*Phorphyromonas gingivalis, Treponema denticola, Tannerella forsythia, Aggregatibacter, Fusobacterium, and Veillonella*	-	-	2	*↑Tannerella forsythia in subgingival biofilms (DP group of the red-complex bacteria. ↓Aggregatibacter*	Classic periodontopathogens diversity increased in the subgingival niche of periodontitis subjects with diabetes
Shi et al. [27]	Cohort Study	32	Metagenomic shotgun sequencing	*Porphyromonas gingivalis, Tannerella forsythia, Treponema denticola, Fusobacterium nucleatum, Campylobacter rectus, Prevotella intermedia, Prevotella nigrescens*	HbA1c	Gingival index and recession, attachment level, pocket depth and bleeding on probing	2	Butanoate metabolism pathway was enriched in the periodontal healthy state in Type 2 DM, but not in No Diabetes.	Type 2 DM patients are more susceptible to shifts in the subgingival microbiome toward dysbiosis in developing periodontitis.
Farina et al. [68]	Case-control study	12	High-resolution whole metagenomic shotgun sequencing	-	HbA1c	Bleeding on probing	2	↑*Anaerolineaceae bacterium* oral taxon 439 in diabetic subjects with moderate to severe periodontitis vs patients without history of periodontitis.	The presence of type 2 Diabetes Mellitus and/or periodontitis were associated with a tendency of the subgingival microbiome to decrease in richness and diversity.

*rDNA, recombinant deoxyribonucleic acid; HbA1c, hemoglobin A1c; BOP, bleeding on probing; IL-6, interleukin 6; IL-17, interleukin 7; RANKL, receptor activator of NF-κB ligand; DM, diabetes mellitus; PD, probing depth; CAL, clinical attachment loss*.

Despite being extremely relevant, considerable knowledge gaps remain regarding the composition of the subgingival microbial under diabetic conditions, especially using modern methodologies and omics approaches. However, the few studies available in the literature, including the present review, should be considered to support future investigations and larger studies.

## Salivary Microbiome Affected By Diabetes Mellitus

Saliva is a complex fluid composed of the secretions from the minor and major salivary glands, mucosal transudations, serum, among others [74]. In the last two decades, saliva has become the focus of a great number of studies, being adopted in diagnostics of oral and systemic diseases [75] because samples can be easily and non-invasively collected [76]. Saliva contains numerous active biomolecules, its microbiome is from various niches in the oral cavity, and appears to be representative of the overall oral microbiome [77]. An analysis of the salivary microbiome is required to better understand how it relates to both diabetes mellitus and periodontitis status.

Oral microbial diversity appears to decrease in patients with type 2 diabetes and increase with the progression of periodontitis compared with periodontally healthy controls [78]. This could be explained by the following two different mechanisms. First, elevated glucose levels in the saliva of subjects with type 2 diabetes mellitus and pre-diabetes could impact the oral environment, enhancing the growth of certain bacterial species at the expense of others. Second, mouth dehydration, usually associated with type 2 diabetes mellitus, could result in microbial diversity reduction [59, 78–80].

Corroborating these data, Pirih et al. [81] assessed individuals with metabolic syndrome and type 2 diabetes mellitus in comparison to healthy patients. The data showed that the salivary microbiome in health was more diverse than the metabolic syndrome group. In addition, the metabolic syndrome periodontitis group displayed a higher abundance of *Tannerella forsythia*. In a different study, higher levels of *T. forsythia, P. gingivalis*, and *F. alocis* were reported in patients with gestational diabetes [82].

Similar results were found by Saeb et al. [59] where a reduction of biological and phylogenetic diversity in the oral microbiota was apparent in type 2 diabetes mellitus and pre-diabetes in comparison with normoglycemic individuals. Janem et al. [83] found a tendency for lower diversity scores in type 2 diabetes mellitus compared to obese groups. Some variation was noted at the genus level where *Haemophilus, Alloprevotella, Pseudomonas*, and *Lautropia* were reduced in diabetes mellitus, while *Fretibacterium* was increased. Norrman et al. [84] did not find differences between the groups in number of periodontal pathogens; however, it is important to note that the patients from this study included liver transplant recipients with type 2 diabetes mellitus, using several systemic medications, such as analgesics, immunosuppressors, cyclosporine, and corticosteroid.

Omori et al. [85] showed no significant differences in the alpha diversity of the salivary microbiota between elderly patients with type 2 diabetes mellitus and control groups. At the genus level, however, an increased abundance of *Rothia, Faecalibacterium*, and *Selenomonas* was observed, as well as a decreased abundance of *Prevotella, Porphyromonas*, and *Neisseria* in the diabetic groups compared to the control group.

Similarly, another study showed that the proportions of *Lactobacillus paraplantarum* and *Acinetobacter nosocomialis* were relatively higher in the periodontitis with the type 2 diabetes mellitus group compared to patients with periodontitis only. In addition, higher proportions of *Prevotella copri, Ralstonia pickettii, Alloprevotella rava, Treponema medium, Faecalibacterium prausnitzii, Eubacterium sulci*, and *Acinetobacter nosocomialis* were found compared to the diabetic group [79]. Interestingly, the proportions of *Streptobacillus moniliformis, Streptococcus mutans*, and *Prevotella jejuni* were significantly higher in the group of patients with type 2 diabetes mellitus that received treatment with Metformin [79]. These data suggest that, after effective glycemic control, the salivary microbial composition of periodontitis patients with type 2 diabetes mellitus resembled that of healthy individuals.

Another study compared the salivary microbiome of healthy patients, patients with type 2 diabetes mellitus without treatment, and patients with diabetes treated with metformin or a combination of insulin and other drugs [80]. This study found that *Blautia wexlerae, Lactobacillus fermentum, Nocardia coeliaca, and Selenomonas artemidis* exhibited a relatively higher abundance in the patients without treatment compared to healthy and the diabetic treatment groups. However, diabetic patients without treatment showed increased severity of periodontitis [80], which could account for the differences in microbial composition.

The differences between the microbiome found in the studies could be justified based on several factors such as: 1) different methods used to assess the microbiome, 2) the association with other systemic diseases, 3) type 2 diabetes mellitus controlled by medication, 4) poorly controlled type 2 diabetes mellitus, 5) the age of the patients, 6) periodontitis classification, 7) sample size, and/or 8) geographic location. Although there is still no consensus on the microbiome in patients with type 2 diabetes mellitus and periodontitis, it is clear that the combined effects of diabetes mellitus and periodontitis on the changes in the salivary microbial composition were significantly greater than that of diabetes mellitus alone, suggesting that periodontitis-related parameters are the main factors influencing the salivary microbial composition [86]. A summary of studies examined for this review is shown in Table 2.

**Table 2:** Salivary microbiome affected by diabetes mellitus.

**References**	**Study design**	**Population**	**Diabetes type**	**Other systemic condition**	**Methods**	**Salivary markers**	**Genus/Species identified**	**Outcomes in diabetic groups**
Janem et al. [83]	Cross-sectional	49	2	Child obesity	ELISA and 16S rRNA	Acidity, CRP, Nitric Oxide, IL-1β, Glucose	*Haemophilus, Alloprevotel, Cardiobacterium, Pseudomonas, Lautropia, Corynebacterium, Scardovia, Fretibacteirum*	↑ Fretibacterium, ↓*Haemophilus*, ↓*Alloprevotel*, ↓*Pseudomonas*, and ↓*Lautropia*
Sabharwal et al. [78]	Cross-sectional	143	2	None	16S rDNA	No markers	88 Genus	Reduceddiversity
Yang et al. [80]	Cross-sectional	102	2	None	16S rDNA	No markers	43 Genus	↑*Blautia wexlerae*, ↑*Lactobacillus fermentum*, ↑*Nocardia coeliaca* ↑*Selenomonas artemidis*
Omori et al. [85]	Case-control	84	2	None	16S rRNA	No markers	127 Genus	↑*Rothia*, ↑*Faecalibacterium*, ↑*Selenomonas*, ↓*Prevotella*, ↓*Porphyromonas* ↓Neisseria
Pirih et al. [81]	Cross-sectional	32	2	Metabolic syndrome	16S rDNA	No markers	98 Novel species	↑*Tannerella forsythia* ↑phylum *Spirochaetes*, ↑genus *Treponema*
Sun et al. [79]	Cross-sectional	133	2	None	16S rDNA	No markers	322 Genus	(DM + P) group compared to periodontitis only: ↑*Lactobacillus paraplantarum, ↑Acinetobacter nosocomialis* (DM + P) group compared to diabetes only: ↑*Prevotella copri, ↑Ralstonia pickettii, ↑Alloprevotella Rava, ↑Treponema medium, ↑Faecalibacterium prausnitzii, ↑Eubacterium sulci ↑Acinetobacter nosocomialis*,
Belstrøm [86]	Review	Overall: 355	2	Gestational diabetes, child and adult obesity	16S rRNA and PCR	No markers	-	*↓Bacterial diversity, ↑P. gingivalis, ↑T. forsythia, ↑F. alocis* (Minor differences in children with T2D, compared to obese and healthy controls)
Norrman et al. [84]	Cross-sectional	84	2	Liver transplant	ELISA	IgA, IgG, IgM, albumin, total protein, TNF-α, IL-1β, MMP-8, TIMP-1	*Candida, Prevotella intermedia, Micromonas micros, Tannerella forsythia, Porphyromonas gingivalis*, and *Parvimonas Micra*	No difference between groups was found in the microbial counts and salivary biomarker levels

*IL, interleukin; MCP-1, monocyte chemoattractant protein 1; MIP-1β, macrophage inflammatory protein 1β; G-CSF, granulocyte-colony stimulating factor; GM-CSF, granulocyte-macrophage colony-stimulating factor; TNFα, tumor necrosis factor-α; IFNγ, interferon-γ (IFNγ); CRP, C-reactive protein; A, G, and M, albumin, total amount of protein, and the cytokines tumor necrosis factor (TNF)-α and interleukin (IL)-1β, as well as matrix metalloproteinase (MMP)-8, tissue inhibitor of MMP (TIMP)-1, and the molar ratio between MMP-8 and TIMP-1; Ig: immunoglobulins; MMP, matrix metalloproteinase; TIMP-1, tissue inhibitor of matrix metalloproteinase; 1,5-AG, anhydroglucitol; TD2: type 2 diabetes*.

Well-designed longitudinal studies are needed to uncover if salivary microbiome changes precede clinical signs of disease, which would enable the use of salivary microbiome signatures for each disease and its diagnosis and risk assessments [78].

## Conclusion

Diabetes mellitus leads to taxonomic differences of microbial species, but little is known about the direct effects of this metabolic disorder on the subgingival and salivary microbiota. Given the uncertainty as to whether the changes reported are due exclusively to hyperglycemia or predominantly, from periodontal inflammation, more studies need to be carried out not only to help answer these questions but mainly to support the development of new periodontal treatments in patients with uncontrolled diabetes.

## Author Contributions

DS, SM, and MC conducted bibliographic research and manuscript writing. BB, RL, and FP contributed to the writing and guided the manuscript. All authors contributed to the article and approved the submitted version.

## Conflict of Interest

The authors declare that the research was conducted in the absence of any commercial or financial relationships that could be construed as a potential conflict of interest.

## Publisher's Note

All claims expressed in this article are solely those of the authors and do not necessarily represent those of their affiliated organizations, or those of the publisher, the editors and the reviewers. Any product that may be evaluated in this article, or claim that may be made by its manufacturer, is not guaranteed or endorsed by the publisher.
